# Transcriptional and Hormonal Regulation of Weeping Trait in *Salix matsudana*

**DOI:** 10.3390/genes8120359

**Published:** 2017-11-30

**Authors:** Juanjuan Liu, Yanfei Zeng, Pengcheng Yan, Caiyun He, Jianguo Zhang

**Affiliations:** 1 State Key Laboratory of Tree Genetics and Breeding, Key Laboratory of Tree Breeding and Cultivation of the State Forestry Administration, Research Institute of Forestry, Chinese Academy of Forestry, Beijing 100091, China; liujj@caf.ac.cn (J.L.); zengyf@caf.ac.cn (Y.Z.); hecy@caf.ac.cn (C.H.); 2 Collaborative Innovation Center of Sustainable Forestry in Southern China, Nanjing Forestry University, Nanjing 210037, China; 3 Beijing Key Laboratory of Cloud Computing Key Technology and Application, Beijing Computing Center, Beijing 100094, China; yanpc@bcc.ac.cn

**Keywords:** phytohormone, gibberellin, auxin, weeping, transcription factor, *Salix matsudana*

## Abstract

*Salix matsudana* is a large and rapidly-growing tree, with erect or spreading branchlets (upright willow). However, *S. matsudana* var. *pseudomatsudana* is one of the varietas, with pendulous branchlets (weeping willow). It has high ornamental value for its graceful pendulous branches. In order to study the molecular basis for this weeping trait, leaves and stems collected at different developmental stages were analyzed using RNA-seq coupled with digital gene expression. Although weeping trees are used worldwide as landscape plants, little is known about the genes that control weeping. Our growth results indicated that branches in weeping willow developed and elongated throughout all developmental stages, but branches in upright willow grew rapidly in the initial stages and then grew slowly and began shoot branching in the middle stages. A total of 613 hormone-related genes were differentially expressed in willow development. Among these, genes associated with auxin and gibberellin (GA) were highly likely to be responsible for the weeping trait, and genes associated with auxin and ethylene probably play crucial roles in shoot elongation. The genes with differential expression patterns were used to construct a network that regulated stem development, and auxin-related genes were identified as hub genes in the network in the weeping willow. Our results suggest an important role of gibberellin and auxin in regulating the weeping trait in *Salix matsudana*. This is the first report on the molecular aspects of hormonal effects on weeping trait in willow using transcriptomics and helps in dissecting the molecular mechanisms by which the weeping trait is controlled.

## 1. Introduction

*Salix matsudana* is native to northeastern China and is widely cultivated in China. The species is a large, deciduous, rapidly growing tree, with erect or spreading branchlets [1]. The Flora of China recognizes three varieties within the species *S. matsudana*, *S. matsudana* var. *pseudomatsudana*, *S. matsudanavar.* var. *anshanensis* and *S. matsudanavar.* var. *matsudana* [2]. However, among the three, only the branchlets of *S. matsudana* var. *pseudomatsudana* are pendulous. As a result of the graceful weeping branches and twigs, the Chinese willow has been introduced into many places, including Australia, Europe and North America, as an ornamental and landscape tree. Having a specific architecture, the pendulous-branched (weeping) form has received increasing attention [3,4,5].

The weeping trait may be attributed to either aberrant gibberellin signaling and/or alterations in wood composition [3,6]. In Japanese cherry, gibberellic acid (GA_3_) promotes internodes in the elongation zone in growing branches but the role of the auxin indole-3-acetic acid (IAA) remains unclear [3]. However, IAA is essential for the maintenance of GA_1_ levels in elongating internodes in pea [7]. Weeping branch internodes are longer than those in upright branches in peach, suggesting a possible up-regulation in GA biosynthesis or response [6,8]. Moreover, it has been reported that GA alone [3], or auxin with GA [9,10] affect(s) the development of secondary xylem. It seems likely that GA_3_ affects relative timing of shoot elongation and the formation of lignified secondary xylem [3,11]. Taken together, these results suggest that the weeping branch may be caused by early failure of a hormone-control system associated with GAs.

In addition, an altered gravitropic response may also be responsible for the weeping type of tree. Tension wood is capable of generating a tensile force, which results in negative tropism of the stem [10,12]. Tension wood is formed in secondary xylem in branches of the upright type of Japanese cherry and not in the weeping type [13]. At the transcriptional regulation level, numbers of genes are differentially expressed in tension wood, including large hormone-related (such as auxin, ethylene, and GA) genes [14]. However, shoot-bending does not cause changes in cytokinin and auxin in Japanese morning glory [15]. To date, several transcription factors (TFs) have been specifically implicated in the weeping phenotype [6]. Kitazawa et al. found that *SCARECROW* (*SCR*), which belongs to the GRAS family of TFs, is responsible for the weeping phenotype [16].

Based on the current analysis of various weeping plant species, many hormone-related genes and several TFs may be responsible for this growth habit. However, the molecular background of the weeping trait in willow species remains poorly defined. Therefore, how different hormone genes and TFs cooperatively regulate the weeping trait in willow is an important question. In order to understand the genes that play a role in controlling the weeping habit, we used tissue-specific and stage-specific RNA-seq coupled with digital gene expression (DGE) across *S. matsudana* (SM) and one related varietas species (*S. matsudana* var. *pseudo-matsudana*, SMP) with strikingly divergent architecture. Here, we integrated what is known about the hormone genes and TFs that control the weeping trait in willow, focusing on these hormone genes and their interactions. Thus, our data provide valuable information for further dissection of the molecular mechanism of weeping trees.

## 2. Materials and Methods

### 2.1. Plant Materials

Two willows with phenotypic divergence in tree architecture were used in this study. Current-year branch and leaf samples of SM and SMP were harvested in the growing season (from April to October) of 2012 in Beijing, China. Plants at seven developmental stages were labeled as G1 (April 16), G2 (May 9), G3 (June 11), G4 (July 17), G5 (August 15), G6 (September 12), and G7 (October 17). Each branch was divided into two portions (top and basal branches, Figure S1). Thus, randomly-selected healthy leaves and two portions of branches were separately collected. Samples from three individual trees (triplicate biological replicates) were harvested at each stage. Samples were immediately frozen in liquid nitrogen and stored at −80 °C until use.

### 2.2. Branch-Length Measurement and ELISA

During the growing season, the lengths of 20 branches (from base to apex) were measured every month from each three tree in SM and SMP, respectively. Furthermore, samples from leaves and two portions of branches at seven developmental stages were used to determine the content of endogenous phytohormones. The hormones IAA, GA_3_, abscisic acid (ABA) and zeatin riboside (ZR) were extracted and purified according to Teng et al. [17]. The ELISA kits used for estimation of the hormone levels came from China Agricultural University (Beijing, China). One-way analysis of variance and the least significant difference (LSD) test were used to determine significant differences in branch length and hormone content. Statistical significance was set at *p* = 0.05.

### 2.3. Sample Preparation and RNA Isolation

Based on the growth measurements and hormone contents, 4 samples of leaves and branches in two willows (SML, SMB, SMPL and SMPB) were used for transcriptome analysis. Each sample at four development stages (G1, G2, G4, and G6) were pooled to provide a broad gene library. Meanwhile, 22 samples of the three tissues at the four stages were collected for DGE analysis (Table 1). Among these, because the branches at G1 were too short, we regarded the top and basal branches as the same sample. For each sample, equal numbers of leaves and branches from three individual trees were pooled as one biological sample for RNA preparation. Total RNA was isolated using the TRIZOL reagent (Invitrogen, Carlsbad, CA, USA) as per the manufacturer’s instructions. The RNA integrity was confirmed using an Agilent Bioanalyzer 2100 (Agilent Technologies, Santa Clara, CA, USA).

### 2.4. Library Preparation for Transcriptome Sequencing, Assembly, and Annotation

After extracting total RNA from 4 samples, the poly (A) mRNA was isolated using beads with oligo (dT) and then interrupted into short fragments. The fragments were purified, followed by end repair, adenylation, adaptor ligation, and PCR amplification. Then, the library was sequenced by the Illumina HiSeq platform (Illumina, San Diego, CA, USA). Clean reads were obtained from the raw data by removing those containing adapter or ploy-N, as well as low-quality reads. Finally, *de novo* transcriptome assembly was carried out with Trinity [18] to obtain unigenes. Gene function was annotated based on unigenes and protein databases (Nr, Nt, Swiss-Prot, KEGG, GO, Pfam, and COG). KOBAS (KEGG Orthology Based Annotation System, Peking University, Beijing, China) software was used to assess the statistical enrichment of differentially expressed genes (DEGs) in KEGG pathways [19]. The raw sequences have been deposited into the Sequence Read Archive (SRA) of NCBI with accession number SRP102505.

### 2.5. Library Preparation for DGE Sequencing

The total RNA from the 22 samples was used for mRNA capture with magnetic oligo (dT) magnetic beads. After adding fragmentation buffer, the mRNA was interrupted into short fragments (~200 bp). Then, first strand cDNA was synthesized with a random hexamer-primer using the mRNA fragments as templates. Buffer, dNTPs, RNase H, and DNA polymerase I were added to synthesize the second strand. The double strand cDNA was purified with magnetic beads. End reparation and 3’-end single nucleotide A (adenine) addition was then performed. Finally, sequencing adaptors were added to the fragments, which were enriched by PCR amplification. During the quality-control step, an Agilent 2100 Bioanalyzer (Agilent Technologies, Santa Clara, CA, USA) and the ABI StepOnePlus Real-Time PCR System were used to qualify and quantify the sample library. The library products were sequenced on an Illumina HiSeq2000. The raw reads have been submitted to the SRA with accession number SRP102498.

### 2.6. Aligning DGEs to the Reference Transcriptome Data and Screening for Differentially Expressed Genes

All tags of the 22 DGE libraries (except low-quality tags, empty tags, and tags with only one copy number) were mapped to our reference transcriptome generated by RNA-seq using RSEM software [20]. No more than two mismatches were allowed in the alignment. The expression of each gene was normalized to the FPKM (expected number of Fragments Per Kilobase of transcript sequence per Million base pairs sequenced) to allow comparison among different samples. Differential expression analysis of two samples was performed using the DEGseq R package. The *p*-value was adjusted using the q-value [21]. A q-value ≤ 0.005 and an absolute value of the log_2_ (fold change) ≥1 were set as the threshold for significantly differential expression.

### 2.7. Gene Validation and Quantitative Real-Time PCR (qRT-PCR)

Ten selected genes with potential roles in hormone metabolism and transcription factors were confirmed by qRT-PCR. The gene-specific primers (Table S1) were designed to correspond to the conserved regions of willow cDNA sequences from the database. Each qRT-PCR reaction was repeated three times. The gene-expression levels were calculated relative to the expression of the actin gene, a reference gene [22], using the 2^−ΔΔCt^ method.

## 3. Results

### 3.1. Variation of Growth and Hormone Content during Development

**Figure 1:**
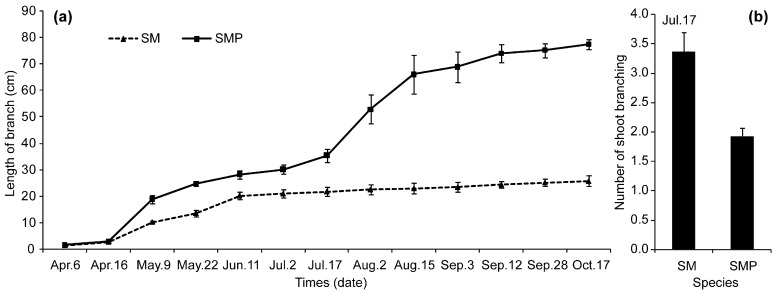
Growth (**a**) and shoot branching numbers (**b**) variation during different developmental stages. SM and SMP represent *S. matsudana* and *S. matsudana* var. *pseudo-matsudana*.

To better understand the growth patterns of the two willows, the lengths of current-year branches were measured from April to October (Figure 1a). Plants began to sprout on April 6, and grew rapidly from April 16 to June 11. As the branches developed after June 11, the speed of growth declined slowly in SM until the late stages of development. Meanwhile, some branches stopped growing and others (56.7%) germinated several axillary buds that developed into new branches from July 17 (Figure S1). Although these axillary buds developed into new branches, they still grew slowly. In addition, the number of shoot branching in SM was greater than those in SMP at July 17 (Figure 1b). In contrast, branches did not stop growing and some (36.7%) germinated axillary buds from July 17 in SMP. All branches maintained rapid growth from June 11 that continued between July 17 and August 15.

**Figure 2:**
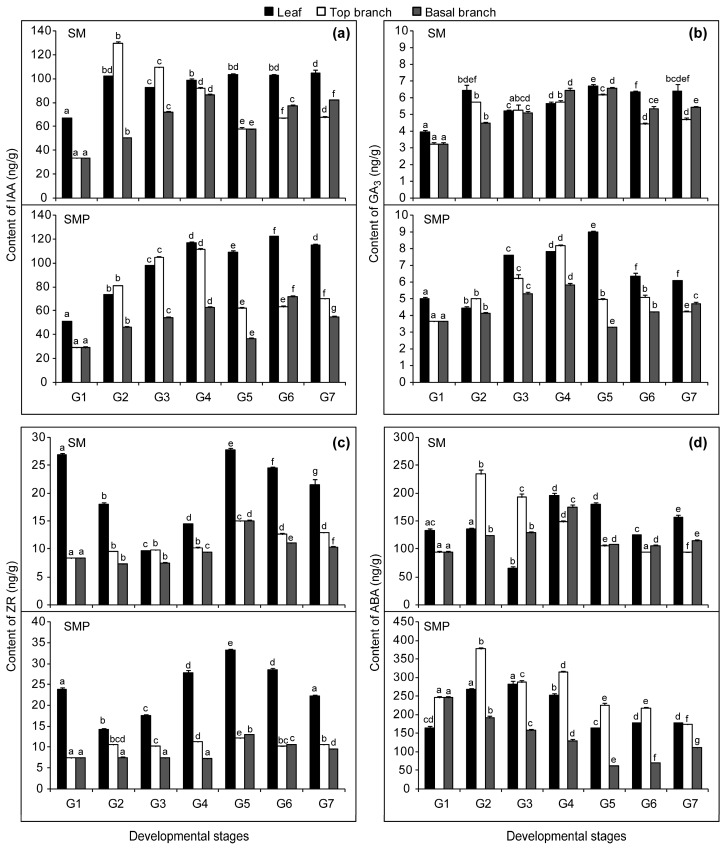
Changes in endogenous hormone content during different developmental stages and in tissues: (**a**) indole acetic acid (IAA); (**b**) gibberellic acid (GA_3_); (**c**) zeatin riboside (ZR) and (**d**) abscisic acid (ABA). Leaf, top branch, and basal branch represent the three tissues, and G1-G7 represent the seven developmental stages. SM, *S. matsudana*; SMP, *S. matsudana* var. *pseudo-matsudana*. Hormone concentrations in fresh weight. Bars represent SE (n = 9). Different letters on columns with the same pattern indicate differences at *P*<0.05 according to the LSD test.

Based on our growth results, three tissues at seven developmental stages (G1–G7) were selected for the determination of endogenous hormone content (Figure 2). The maximum peak concentrations of IAA in the top branches appeared at G2 and G4 (Figure 2a). The first peak appeared in the basal branches at G4 and the second peak at G6. The first peak appeared in leaves at G2 in SM and at G4 in SMP, and both maintained higher IAA concentrations after G4. At G1 and G2, the IAA concentrations in the leaves and top branches in SM were higher than those in SMP, while conversely, from G4 they were lower than those in SMP. Throughout development, the IAA concentrations in the basal branches of SM were always higher than those in SMP.

The maximum peak concentration of GA_3_ in all three tissues appeared at G5 in SM, while that in SMP appeared at G4 in branches (Figure 2b). Moreover, the maximum peak concentration of ZR in all three tissues appeared at G5 in SM and SMP (Figure 2c). In leaves, GA_3_ and ZR concentrations in SM were lower than those in SMP after G2, consistent with the changes in IAA concentration. The maximum peak concentration of ABA in top appeared at G2 (Figure 2d). From G1 to G6, the GA_3_ and ABA concentrations in the top branches were higher than those in the basal branches in SMP. However, in SM, this was only found at early developmental stages. Therefore, these changes prompted us to study the molecular mechanisms at the transcriptome level. Finally, samples at G1, G2, G4 and G6 were used for further transcriptome and DGE analysis.

### 3.2. Transcriptome and Digital Gene Expression Sequencing in Willows

**Figure 3:**
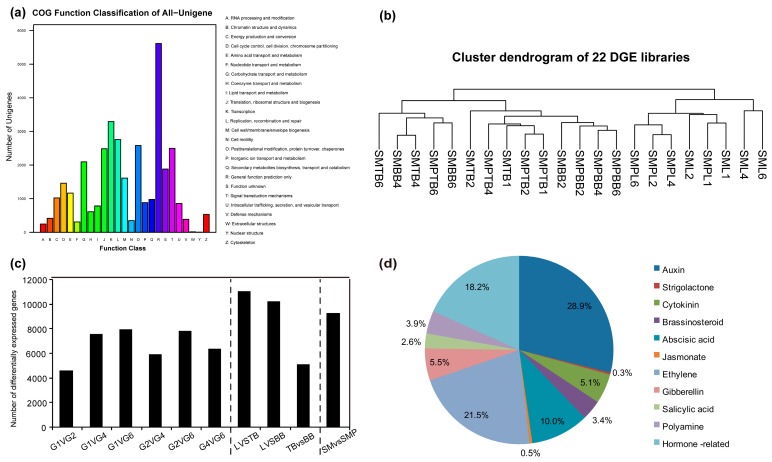
Global characterization of gene expression patterns among three tissues at four developmental stages. (**a**) Cluster of Orthologous Group (COG) classification of unigenes. A total of 17, 013 unigenes were assigned to the COG database. The horizontal axis is the 26 COG categories and the vertical axis is the number of unigenes in each category. (**b**) Cluster dendrogram showing relationships of gene expression in different tissues and species at four developmental stages. Branch length indicates degree of variance; sample names defined in Table 1. (**c**) Histogram analyses of differentially expressed genes between developmental stages and tissues. L, leaf; TB, top branch; BB, basal branch; SM, *S. matsudana*; SMP, *S. matsudana* var. *pseudo-matsudana*. (**d**) Classification of identified genes based on hormones.

To study the molecular mechanisms in weeping willow, we constructed and sequenced four RNA-seq libraries and 22 DGE libraries from SM and SMP. In each transcriptome library, 21.9–25.8 million clean reads were obtained (Table 1). Overall, 73,141 unigenes were expressed with a total length of 43,214,574 bp and a mean length of 591 bp (Figure S2a). Next, we investigated the functional annotations of the unigenes in each sample. A total of 53,620 unigenes (73.31% of all unigenes) provided significant BLAST hits. Among these, 17,013 unigenes were aligned to the COG database in order to predict and classify possible functions (Figure 3a). Among the 26 COG categories, the largest was the cluster of general function prediction only (5,614, 33.0%), followed by transcription (3,293, 19.4%), replication, recombination and repair (2,759, 16.2%), posttranslational modification, protein turnover, chaperones (2,581, 15.2%) and signal transduction mechanisms (2,498, 14.7%). These results suggested that genes associated with transcription and signal transduction are highly enriched in willow. In addition, 9,389 unigenes with BLASTx matches were assigned to 265 KEGG pathways (Figure S2b). Among these, unigenes were significantly enriched in translation, carbohydrate metabolism and signal transduction.

In each DGE library, clean reads were mapped to our reference sequences from the RNA-seq based transcriptome (Table 1). A correlation dendrogram showed that data from the top branches clustered with those of the basal branches (Figure 3b), indicating that the top branches retain gene expression patterns similar to the basal branches in both SM and SMP compared to leaves. Then, pairwise comparison of DGE profiles between the same tissues at different development stages (G1, G2, G4 and G6) and those between different tissues (leaves, top branches and basal branches) at the same developmental stage in the two species were analyzed (Figure S3a). Meanwhile, pairwise comparisons between willows in the same tissue and developmental stage were also analyzed. Finally, 15,767 DEGs among the 22 DGE libraries were identified, demonstrating substantial changes in the three tissues during four stages in the two willows (Figure 3c).

To better understand the hormonal control of weeping in willow, we screened hormone-related genes for further comparative analysis (those associated with auxin, cytokinin, strigolactone, ABA, GA, ethylene, brassinosteroid, jasmonic acid, polyamine and salicylic acid). Among these, 1,250 unigenes were associated with hormones, and most were associated with auxin, ethylene and ABA (Figure 3d, Table S2). Finally, 613 genes were differentially expressed in the three tissues at the four stages in willow. The number of DEGs in SM (553) was greater than that in SMP (427). Most of these genes were associated with ethylene (27.7%), auxin (26.2%) and ABA (8.3%) in SM and ethylene (30.4%), auxin (27.9%), and GA (7.5%) in SMP.

### 3.3. Differentially Expressed Genes Involved in Hormone Signal Transduction in Willow

**Figure 4:**
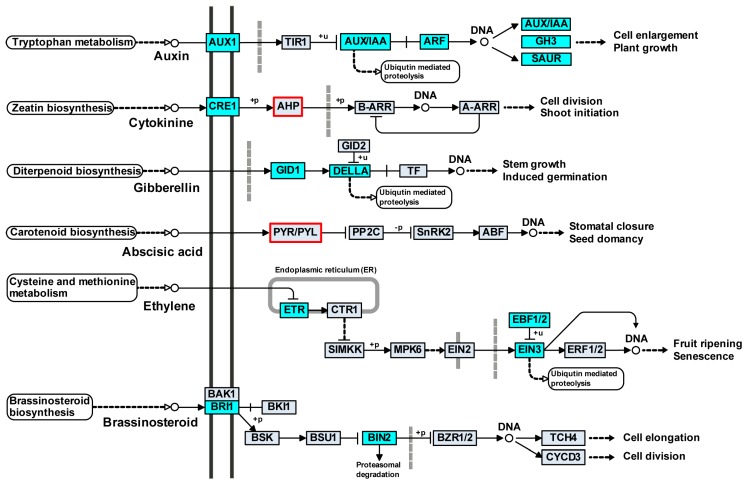
Differentially expressed genes involved in hormone signal transduction pathways in willow. Genes differentially expressed in *S. matsudana* are shown by cyan and red rectangular frames and those in *S. matsudana* var. *pseudo-matsudana* are shown by cyan boxes. AUX1, auxin influx carrier; AUX/IAA, auxin-responsive protein IAA; ARF, auxin response factor; GH3, auxin responsive GH3 gene family; SAUR, SAUR family protein; CRE1, *Arabidopsis* histidine kinase 2/3/4 (cytokinin receptor); AHP, histidine-containing phosphotransfer protein; GID1, gibberellin receptor GID1; PYR/PYL, abscisic acid receptor PYR/PYL family; ETR, ethylene receptor; EBF1/2, EIN3-binding F-box protein; EIN3, ethylene-insensitive protein 3; BRI1, protein brassinosteroid insensitive 1; BIN2, protein brassinosteroid insensitive 2.

To further understand the function of these hormone-related DEGs, KEGG pathway enrichment analysis was performed. The most enriched pathway was plant hormone signal transduction. The DEGs that were significantly enriched in the plant hormone signal transduction pathways were associated with auxin, cytokinin, GA, ABA, ethylene and brassinosteroid in SM, while the genes were linked with auxin, cytokinin, GA, ethylene and brassinosteroid in SMP (Figure 4). The proportions of DEGs involved in auxin and ethylene were remarkably high compared with those associated with cytokinin, GA, ABA and brassinosteroid (Table S3). It is worth noting that the DEGs in the cytokinin and ABA signal transduction pathways were different in SM and SMP. The DEGs were not involved in jasmonic acid and salicylic acid signal transduction pathways in willow.

In the auxin signal transduction pathway, we identified seven auxin influx carrier (*AUX1*), ten auxin response factor (*ARF*) and 19 early auxin response genes (2 *AUX/IAA*, 8 *Gretchen Hagen3* [*GH3*], and nine small auxin-up RNA [*SAUR*]). Most were down-regulated (SM *versus* SMP) at G1, G4, and G6 in all three tissues and at G2 in leaves (Table S3). Among them, *AUX1*, *AUX/IAA*, and *SAUR* genes were down-regulated and *ARF* genes were up-regulated during the growth stages. Four GA receptor *GID1* (*GA-INSENSITIVE DWARF 1*) genes and two *DELLA* proteins were identified. The *GID1* proteins were up-regulated (SM vs SMP) after G4 and the *DELLA* proteins were only differentially expressed at G4 in all three tissues. Furthermore, *GID1* proteins were up-regulated only in the top branches and *DELLA* proteins were down-regulated after G2 in all three tissues.

In addition, three cytokinin receptor (*CRE1*) and one histidine-containing phosphotransfer protein (*AHP*) genes were identified. The *AHP* gene was down-regulated (SM vs SMP) after G2, specifically differentially expressed in SM, and down-regulated during the growth stages in all three tissues (Table S3). Compared with leaves, most of the *CRE1* genes were up-regulated in stems. Only one ABA receptor PYR/PYL family gene was identified. The *PYR/PYL* gene was up-regulated (SM vs SMP) in leaves after G4 and specifically differentially expressed in SM. One *BRASSINOSTEROID-INSENSITIVE 1* (*BRI1*) and two *BRASSINOSTEROID-INSENSITIVE 2* (*BIN2*) genes were identified, and most were down-regulated after G2. The expression levels of these genes were relatively higher in stems than in leaves. Seven ethylene receptor (*ETR*), four ethylene-insensitive 3 (*EIN3*) genes and three EIN3-binding F-box proteins (*EBF1/2*) were identified. Most of the *EBF1/2* and *EIN3* genes were up-regulated (SM vs SMP) during the growth stages and the *ETR* genes were not differentially expressed in species and stages.

### 3.4. Genes Involved in IAA and GA Biosynthesis in Willow

**Figure 5:**
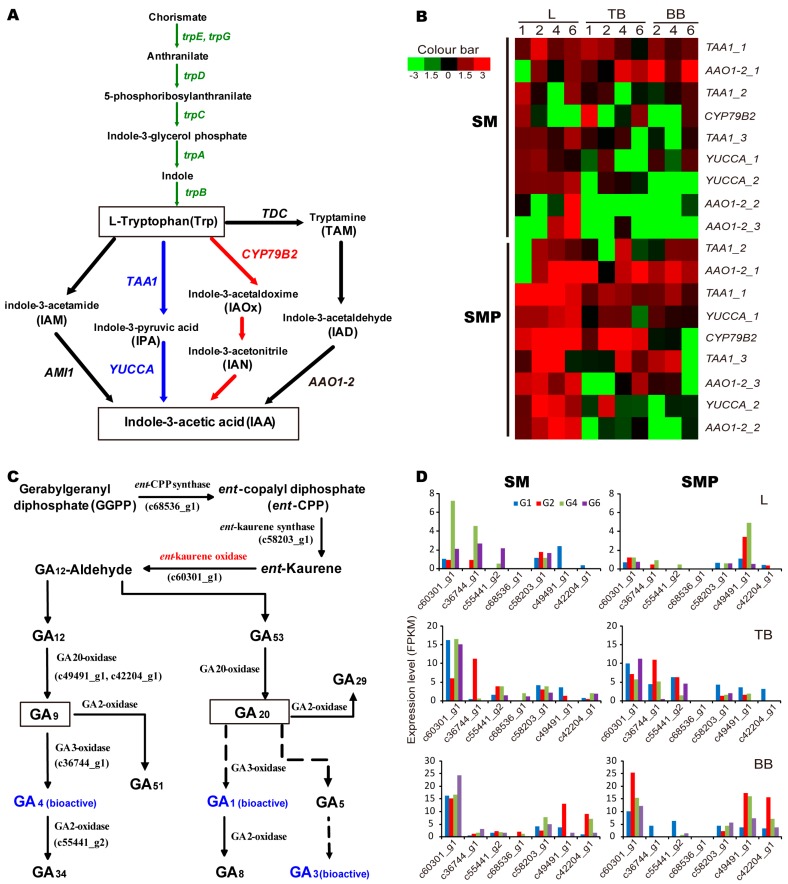
Genes involved in IAA and GA biosynthesis pathways in willow. (**a**) Pathway of IAA biosynthesis. Green arrows, tryptophan synthetic pathway; blue and red arrows, steps for which gene and enzymatic function are known in the tryptophan-dependent IAA biosynthetic pathway; letters in italics, genes involved in the conversion process. (**b**) Heat map of genes involved in IAA biosynthesis during development. Scaled log_2_ expression values are shown from green to red, indicating low to high expression. *TAA1*, L-tryptophan-pyruvate aminotransferase; *AAO1_2*, indole-3-acetaldehyde oxidase; *YUCCA*, indole-3-pyruvate monooxygenase; *CYP79B2*, tryptophan N-monooxygenase. L, leaf; TB, top branch; BB, basal branch; SM, S. matsudana; SMP, S. matsudana var. pseudo-matsudana. (**c**) Gibberellin biosynthesis pathways. (**d**) Expression patterns of genes involved in gibberellin biosynthesis during development.

To explore the molecular mechanisms underlying the actions of IAA and GA in willow development, we analyzed genes encoding the key enzymes involved in all four of the known tryptophan-dependent IAA and GA biosynthesis pathways. First, we found that these genes were involved in two of the four pathways and most were specific to the indole-3-pyruvic acid (IPA) pathway (Figure 5a blue part), while fewer were associated with the indole-3-acetaldoxime (IAOx) pathway, suggesting that this may be a compensatory pathway in willow (Figure 5a red part). Expression analyses showed that L-tryptophan-pyruvate aminotransferase (*TAA1*) and indole-3-acetaldehyde oxidase (*YUCCA*), key enzymes in the IPA pathway, were expressed at higher levels in SMP than in SM (Figure 5b). Tryptophan N-monooxygenase (*CYP79B2*, the key enzyme in the IAOx pathway) showed higher expression levels in leaves and top branches than basal branches in SMP throughout development, but not in SM. Among these, only the *CYP79B2* gene was differentially expressed. We did not find tryptophan decarboxylase (*TDC*) genes in our data, although indole-3-pyruvate monooxygenase (*AAO1_2*) were expressed at higher levels in SMP than in SM. Collectively, our data indicate that the IPA pathway is the major route to the IAA biosynthesis pathway in willow. Then, we found that these genes were involved in two main GA biosynthesis pathways and GA_4_ was the dominant bioactive GA in willow (Figure 5c). Among them, only the *ent*-kaurene oxidase gene (c60301_g1) was differentially expressed between the three tissues in SM and SMP. Expression analyses showed that *ent*-copalyl diphosphate synthase (c68536_g1) was only expressed in the stem tissue of SM (Figure 5d). GA_12_ is converted to the bioactive form GA_4_ by the enzymes GA 20-oxidase and GA 3-oxidase, and the bioactive GA_4_ is catalyzed by GA 2-oxidase. Many of these genes displayed a similar expression pattern: higher abundance in stems than in leaves and higher at G2 than other stages in both SM and SMP.

### 3.5. Transcription Factors in Willow

**Figure 6:**
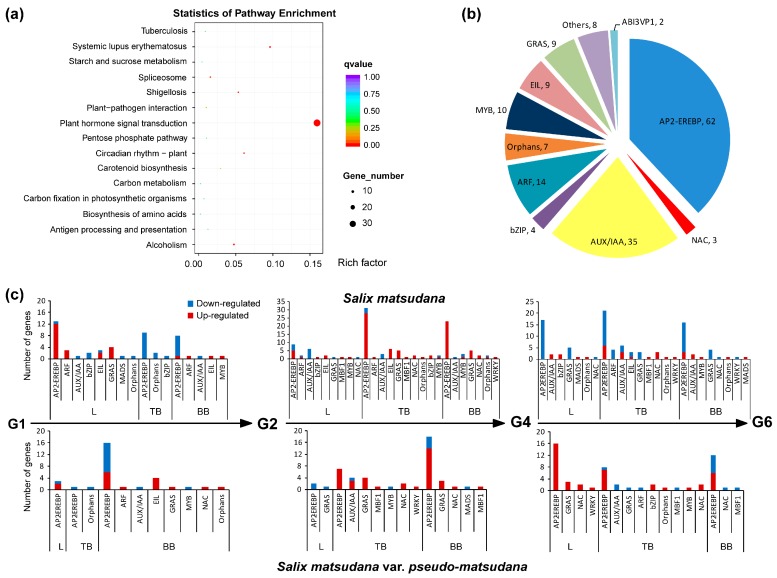
Transcription factors associated with hormones in willow. (**a**) Statistics of pathway enrichment analysis. Horizontal axis, enrichment factor; vertical axis, pathway category. (**b**) Family assignment of hormone-related transcription factors. Number of genes assigned to each family shown after the comma. (**c**) Number of differentially expressed transcription factors at development stages. L, leaf; TB, top branch; BB, basal branch.

Apart from the hormonal control of weeping in willow, a total of 987 (SM, 887; SMP, 661) differentially expressed TFs were found, and they were distributed among 69 gene families (Figure S3b). KEGG pathway analysis revealed that most of these genes were involved in plant hormone signal transduction (Figure 6a). Thus, a total of 163 hormone-related TFs were screened, and assigned into 18 gene families (Figure 6b, Table S3). Among the hormone-related TFs affected in willow, auxin and ethylene clearly dominated. The number of TFs in SM was greater than that in SMP.

To determine the differential expression of 163 TFs by species, we first compared the expression levels between tissues. Leaves showed the highest number of TFs (71), followed by top branches (47) and then basal branches (44). In top branches, members of the AUX/IAA family (c56397_g3, c59114_g3, and c59114_g4) displayed>25-fold up-regulation at G4, and a gene in the GRAS family (c47115_g1) showed 35-fold down-regulation in weeping willow at G6 (Table S3). Then, gene expression levels during development were assessed (Figure 6c). More differentially expressed TFs occurred at G4 than at other stages. Among the TFs that were differentially expressed in stems between stages, most were down-regulated between G1 and G2 in SM, except for the ARF, EIL and MYB family. Meanwhile, the ARF, EIL, NAC and GRAS family genes were up-regulated in SMP. However, between G2 and G4, most of the TFs were up-regulated. In top branches, members of the GRAS family (c25921_g1, c62314_g1, c47115_g2, and c47115_g1) and MBF1 family (c54190_g1) displayed >16-fold up-regulation (G4 vs G2) in SM.

### 3.6. Co-Expression of Hormone-Related Genes in Willow

**Figure 7:**
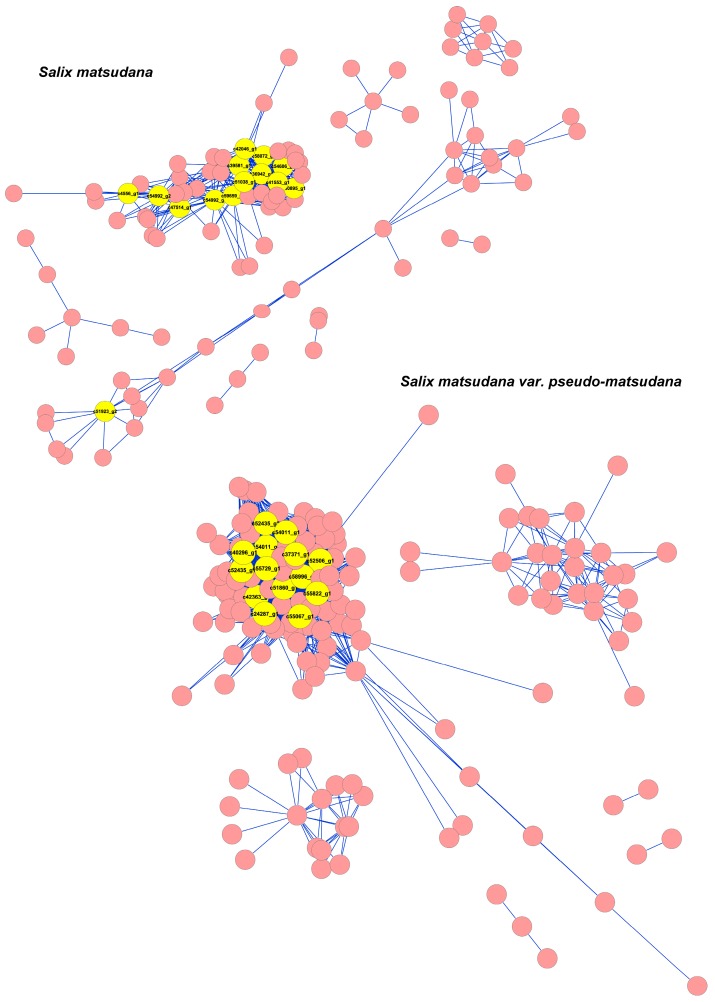
Co-expression networks of hormone-related genes in willow. Each node (circles) represents a gene and edges (connecting lines) between genes represent co-expression correlations. Hub genes are labeled as larger yellow circles. One hundred sixty-seven genes with an edge weight >0.4 were visualized using Cytoscape software.

We used an alternative analysis tool, WGCNA (an R software package for weighted correlation network analysis, Los Angeles, CA, USA), which is based on a systems biology approach aimed at understanding networks rather than individual genes [23,24]. Co-expression networks were constructed on the basis of hormone-related genes in SM and SMP (Figure 7, Table S4). Hub genes tended to have high connectivity in the network. Strikingly, many of the hub genes were auxin-related genes in weeping willow (Table S4): two auxin responsive *GH3* genes, three auxin-binding proteins (*ABP20*/c37371_g1, c51860_g1; *ABP19A*/c54011_g2), the auxin-responsive protein *SAUR20* and the thermospermine synthase *ACL5* gene involved in polar auxin transport. The hub gene with the highest edge number (58 edges) in weeping willow was the auxin-binding protein *ABP20*, a probable receptor for the growth-promoting hormone auxin. This gene was highly expressed at stages G1–G4 in SMP, but at G1 and G2 in SM leaves. In upright willow, many of the hub genes were auxin- and ethylene-related (Table S4), including those for three proteins in the auxin-responsive family, auxin-induced in root cultures protein 12 (*AIR12*) and four ethylene-responsive transcription factors (*ABR1*-like/c42046_g1 and c47514_g1; *ERF110*/c58072_g1; *ERF019*/c36942_g1). The hub gene with the highest edge number (33 edges) in upright willow was NAC domain-containing protein 72 (*NAC072*), which is strongly induced by ABA. Other highly connected hub genes were cytokinin hydroxylase (c51038_g1), cytokinin dehydrogenase 1 (c59659_g1) and probable strigolactone esterase D14 (c51923_g2).

### 3.7. Validation of Candidate Hormone-Related Genes by qRT-PCR

To verify the expression levels of DEGs identified by RNA-Seq and DGE, we performed qRT-PCR assays using samples that were collected independently at the same developmental stages as those used for the RNA-Seq analysis. Among the ten randomly selected DEGs, all showed the same expression patterns in the qRT-PCR assays as in the RNA-Seq and DGE data (Figure S4). The Pearson correlation coefficient between qRT-PCR and RNA-Seq data was 0.739 (*p* < 0.01).

## 4. Discussion

### 4.1. Genes Associated with Hormone Synthesis in Willow

In this study, we provide a comprehensive analysis of plant hormone genes involved in the control of the weeping trait in willow. Hormones affect which tissues grow upward and which grow downward, leaf formation, stem growth, fruit development and even death. Many hormones have been shown to be essential for the weeping habit, particularly GA and auxin [3,10]. Bioactive GAs are synthesized via complex pathways and regulate stem elongation [7]. We found that GA_4_ was the dominant product in willow; it is known to play a more pivotal role than GA_1_ in controlling shoot elongation [25]. The expression of the GA dioxygenase gene *GA20ox2*, which acts as a limiting step during GA biosynthesis in the control of shoot growth in aspen [25], was higher in the weeping branch before G6. In rice, *GA20ox2*, which encodes a DELLA protein, is expressed in rapidly elongating or dividing tissues [7]. Besides, auxin is predominantly represented by IAA and IAA biosynthesis in plants is fairly complex [26]. Different species may have unique strategies and modifications to optimize their metabolic pathways. Our data showed that the IPA pathway was the major pathway and the IAOx pathway was a compensatory pathway in willow. Among these enzymes, cytochrome P450 (*CYP79B2*) was up-regulated in the top branches of weeping willow at G2. Overexpression of *CYP79B2* in *Arabidopsis* probably leads to overproduction of IAOx, thus increasing the flux of IAOx to IAA biosynthesis [27].

### 4.2. Genes Associated with Hormone Signal Transduction in Weeping Branch Control

A total of 1,250 hormone-related genes were found, of which 613 were differentially expressed between any two stages, tissues, or species. Among these, genes associated with hormone signal transduction were identified: 36 auxin, four cytokinin, six gibberellin, one ABA, 14 ethylene and three brassinosteroid genes. *AUX1* genes, which are expressed in the surface layer of shoot apical meristem [28], were up-regulated in top branches compared with other tissues. In top branches, the high expression of *AUX1* genes was mainly at early stages (G1 and G2) in upright willow, but occurred throughout development in weeping willow. Also, *AUX1* genes were up-regulated in weeping branches. However, Bennett et al. reported that AUX1 regulates root curvature [29]. Furthermore, we found six members of the ARF family (ARF1, 3, 5, 9, 11, and 18), but there was no notable expression difference between weeping and upright willows. *ARF1* and *ARF3* were down-regulated in leaves; *ARF1* regulates leaf senescence [30] and *ARF3* functions in leaf polarity specification [31]. Moreover, *ARF5* was up-regulated in stems; it plays key roles in auxin-mediated morphogenesis, promoting shoot formation in *Arabidopsis*, and secondary xylem development in *Populus* [32,33]. High expression of *ARF5* was displayed at G2 in weeping branches but only in the basal branches of upright willow. In the weeping willow, *ARF9* expression was higher in basal branches than in top branches; Roberts et al. showed that *ARF9* is involved in tropic transduction/response mechanisms [34]. In addition, *ARF11* was only differentially expressed in upright willow and *ARF18* was up-regulated in stems; their functions await further study.

In the regulation of plant development and growth, auxin can rapidly and transiently induce the expression of three groups of genes: SAUR, GH3, and AUX/IAA. SAUR family genes (*SAUR22, 23* and *32*) were up-regulated in weeping willow. Chen et al. revealed that SAUR19-24 function as positive effectors of cell expansion [35], and Xie et al. demonstrated that *SAUR32* induces short, hookless hypocotyls when overexpressed in *Arabidopsis* [36]. In addition, most GH3 family genes were up-regulated in weeping willow. Among them, high expression of the *GH3.2* gene was displayed in top branches at G6, but that of *GH3.6* occurred at G1. *GH3.6* overexpression causes dwarfism, inhibits cell growth and loosens the cell wall, and *GH3.2* overexpression produces a dwarf phenotype [37]. Our results showed that AUX/IAA (*IAA13* and *AUX22D*) were up-regulated in weeping branches at later developmental stages (G4 and G6). However, Weijers et al. have demonstrated that IAA13 is involved in embryonic root initiation [38]. The above genes are associated with the auxin signaling pathway, indicating that *AUX1* and *ARF9* possibly play a role in the tropic response and *ARF5* in shoot growth of the weeping willow. Taken together, we thus hypothesized that auxin has signaling or signal-integrating properties pertinent to the regulation of the weeping trait.

Ethylene is well known to interact with auxin signaling, and is induced during the formation of tension wood. Four ethylene receptor genes (*ETR1*, *ETR2*, *ERS1*, and *ERS2*) have been identified. However, these genes were not differentially expressed between willows and stages. Agarwal et al. have shown that ethylene receptors play a role in senescence and ripening in horticultural crops [39]. Furthermore, *EBF1* was mostly down-regulated in the top branches of weeping willow, while *EBF2* was not differentially expressed between willows. EBF1 and EBF2 have recently been shown to function in ethylene perception by regulating EIN3 turnover [40]. Our results showed that *EIN3* was down-regulated in weeping willow. Overexpression of *EIN3* results in a slightly short shoot phenotype in rice [41]. Most of the *EIN3* was more highly expressed in stems, showing that it plays a more important role in shoot elongation. This indicated that the *EIN3* possibly plays a role in regulating shoot elongation in weeping willow.

GA is an important hormone for growth and development, such as shoot elongation. GA signaling relies on the perception of the hormone by its receptor GID1. Two *GID1* orthologs (*GID1B* and *GID1C*) were differentially expressed in willows during the developmental stages. Griffiths et al. demonstrated that *GID1A* and *GID1C* play a major role in promoting stem elongation, but *GID1B* has a minor influence in *Arabidopsis* [42]. However, the unique characteristic of *GID1B* in *Arabidopsis* in inflorescence stems may be advantageous to plants, because it prevents them from becoming slender [43]. Our result showed that *GID1C* did not change significantly in branches during development, but *GID1B* was up-regulated in upright willow after G4 and high expression was displayed in weeping branches at G1 and G6 and in upright branches after G4, implying that *GID1B* promotes stem elongation in weeping willow at specific growth stages and prevents upright willow from becoming slender after the shoot branching stage (G4). GID1A–C display functional redundancy in the promotion of stem elongation that is known to be controlled by individual DELLAs [42], which have been proposed to act as repressors of the growth regulation pathway [44]. Also, we found that the DELLA protein *GAI* (*gibberellin insensitive*) of the GRAS family was down-regulated in upright willow. *GAI* is the major repressor of GA-mediated stem growth, and shows the highest preference for *GID1B*, which has a higher affinity for the most active GA_4_ than other GID1s [43,45], consistent with our results for GA synthesis. The emergence of any phenotype(s) depends on the abundance of the remaining receptor and its preference for DELLA proteins at a target site. Here, the high expression of *GAI* in top branches occurred at early developmental stages (G1 and G2), and was subsequently down-regulated (G4 and G6). *GAI* expression was least in the terminal stage (G6). In stem elongation, it has been reported that functional loss of *GAI* leads to a clear slender phenotype [43]. The above findings, combined with the present results of increased *GAI* transcripts in weeping willow while those of *GID1B* decreased, suggested that the interaction of *GID1B* and the DELLA protein *GAI* plays a dominant role in weeping willow.

Cytokinins influence cell division and shoot formation. Most of the AHPs are positive regulators of cytokinin signal transduction. *AHP1* was up-regulated in weeping branches at G2 and G4, although it functions in the root elongation, shoot chlorophyll content, and hypocotyl elongation responses to cytokinin [46]. Furthermore, the abundance of the *CRE1* genes gradually decreased during stem development, but was not differentially expressed in weeping and upright willows. Hutchison et al. discovered that loss-of-function mutations of *CRE1* result in reduced cytokinin sensitivity [46]. In addition, the ABA receptor *PYL9* was up-regulated in leaves only in SM after G4, consistent with the previous report that *PYL9* promotes leaf senescence [47]. Moreover, brassinosteroids are sensed by the membrane receptor kinase BRI1. Here, *BRI1* was up-regulated in leaves after G4 in weeping willow. It has been reported that loss-of-function of *BRI1* leads to severe developmental defects, including extreme dwarfism, altered leaf morphology and delayed senescence [48]. Also, *BIN2* genes were down-regulated in weeping willow, and one of its isoforms (c64152_g1) showed a high abundance in stems from G1 to G6. There is a BIN2-independent regulatory mechanism downstream of BRI1 activation that is crucial for brassinosteroid-regulated growth [49]. Our results suggest that genes in the cytokinin, ABA, and brassinosteroid signal transduction pathways do not play a role in regulating the weeping trait in willow.

### 4.3. Identification of Transcription Factors in Weeping Branch Control

We found 163 hormone-related TFs that were differentially expressed in willow, and most were in the AP2-EREBP, AUX/IAA and ARF gene families. Among them, the abundance of *CIGR1* (*chitin-inducible gibberellin-responsive protein 1*), which belongs to the GRAS family and functions in stress resistance [50], was higher than other TFs. Especially after G4, four *CIGR1* genes were up-regulated in upright stems and extremely highly expressed in the stems of *S. matsudana*, which is a drought tolerance species. Another GRAS family gene, scarecrow-like protein 3 (*SCL3*), enables the regulation of GA feedback [51] and plays a role in endodermal specification, perhaps by regulating the expression of *SCR* or by being regulated by *SCR* [52]. *SCL3* genes were up-regulated in upright stems at G4. Furthermore, *SCR* is responsible for the weeping phenotype in Japanese morning glory [16] and binds to the promoter of *SCL3*. Hormones such as auxin, ethylene and GA have been suggested to mediate responses, but their role and mechanisms of action have not been conclusively established [53,54]. Class I Knotted1-like homeobox (KNOX1) TFs repress GA20ox gene expression and hence GA biosynthesis, thus promoting meristem activity [55]. And we found that the *GA20ox* expression (c49491_g1 and c42204_g1) was low in top branches. Bending and GA stimulation of tension wood formation are sensitive to transcript levels of the KNOX1 homeodomain protein ARK2 in *Populus* [10]. However, *KNOX1* was not differentially expressed between weeping and upright willow but was highly expressed in branches throughout development. Therefore, we speculate that TFs associated with GA may be key for weeping control in willow.

### 4.4. Genes Networks for Weeping/Upright Branch Control

Based on all the above results, we constructed a network for the mechanism in the weeping and upright branches of willow (Figure 7). Auxin- and ethylene-related genes were identified as hub genes operating in the upright willow network. Ethylene is well known to interact with auxin signaling, and ethylene-responsive TFs were up-regulated after G4 and highly expressed in top branches at G6, probably activating stress-responsive genes [56]. Strikingly, other highly connected hub genes were associated with cytokinin and strigolactone. Three classes of phytohormone, auxin, cytokinin, and strigolactone, are central to the control of shoot branching [57]. Our results suggested that auxin, cytokinin, and strigolactone regulate shoot branching in the upright willow, and thus prevent of elongation growth, which is consistent with our previous descriptions of growth.

In weeping willow, auxin-related genes were identified as hub genes in the network. Thus, auxin plays an important role in both weeping and upright willow. Among these genes, *SAUR20* was up-regulated at G4 in leaves and top branches in weeping willow, where they function as positive effectors of cell expansion [35]. Auxin-binding proteins (*ABP19* and *ABP20*) were highly expressed in leaves of weeping willow throughout development while they were highly expressed before G2 in upright willow; they are probable receptors for the growth-promoting hormone auxin and play important roles in expansion and lignification of the cell wall [58]. Also, *ACL5* was up-regulated at G4 in top branches in weeping willow. It has been reported that loss-of-fuction mutants of *ACL5* have severe defects in stem elongation in *Arabidopsis* [59] and show a severe dwarf phenotype [60]. Another gene, *PIN3* (*auxin efflux carrier component 3*), was found in the correlation network of weeping willow but not in that of upright willow. Moreover, *PIN3* expression was up-regulated in weeping willow, especially after G4. It has been reported that PIN3 mediates tropic growth [10,61].

We provide, for the first time, some insights into the molecular mechanisms underlying the hormonal control of growth and development of weeping in willow. During stem development, genes linked to auxin (*AUX1*, *ARF9* and *PIN3*) and GA (*GID1B* and *GAI*), and TFs (*SCL3* and *KNOX1*) associated with GA are probably responsible for the weeping trait, and genes associated with auxin (*ARF5* and *ACL5*) and ethylene (*EIN3*) may play roles in shoot elongation. The reasons for these preferences will be the focus of our future research on GAs and auxin.

## Figures and Tables

**Table 1:** Summary of the DGE sequencing and assembly quality.

Samples	Stages	Tissues	Sample Name	Clean Reads	Total Mapped
*S. matsudana*	G1	Leaf	SML1	6,702,938	82.17%
		Top branch	SMTB1	6,630,850	80.96%
	G2	Leaf	SML2	6,502,380	82.45%
		Top branch	SMTB2	6,135,406	69.76%
		Basal branch	SMBB2	6,470,393	81.08%
	G4	Leaf	SML4	6,816,182	81.31%
		Top branch	SMTB4	6,382,060	78.68%
		Basal branch	SMBB4	6,728,207	78.69%
	G6	Leaf	SML6	5,097,804	74.54%
		Top branch	SMTB6	6,466,563	67.05%
		Basal branch	SMBB6	7,279,661	80.36%
*S. matsudana* var. *pseudo-matsudana*	G1	Leaf	SMPL1	6,157,313	77.55%
		Top branch	SMPTB1	6,490,499	80.22%
	G2	Leaf	SMPL2	6,758,010	82.07%
		Top branch	SMPTB2	5,742,822	80.46%
		Basal branch	SMPBB2	6,274,553	80.46%
	G4	Leaf	SMPL4	6,763,774	82.07%
		Top branch	SMPTB4	7,159,844	81.47%
		Basal branch	SMPBB4	5,637,627	78.12%
	G6	Leaf	SMPL6	6,790,424	80.78%
		Top branch	SMPTB6	7,571,192	80.32%
		Basal branch	SMPBB6	5,907,379	76.53%

## Supplementary Materials

The following are available online at www.mdpi.com/2073-4425/8/12/359/s1. Figure S1: Typical examples of developmental stages of *S. matsudana* and *S. matsudana* var. *pseudo-matsudana*. A schematic of the two portions of branches is shown in the right level. The positions indicated by arrows were sampled. Table S1: Primers used for qRT-PCR. Figure S2: Length distribution of unique-mapped reads of assembled unigenes (a) and KEGG classification of unigenes (b). Figure S3: (a) Histogram of differentially expressed genes in the DGE library. (b) Family assignment of transcription factors (number of genes assigned to each family shown after the comma). Table S2: Statistics of 1,250 genes associated with hormones. Table S3: Expression levels of DEGs involved in hormone signal transduction pathways and differentially expressed transcription factors. Table S4: Details of co-expression networks of hormone-related genes in willow. Figure S4: Quantitative RT-PCR of 10 candidate genes associated with hormones. The relative expression level of each gene is expressed as the fold-change between three tissues and four stages in DGE and qRT-PCR data (expression normalized to the actin gene).
