# Supplementary figures and images for: Transcriptional and Hormonal Regulation of Weeping Trait in Salix matsudana
Source: Genes (Basel). 2017 Nov 30;8(12):359. doi: 10.3390/genes8120359 (PMC5748677; doi:10.3390/genes8120359)

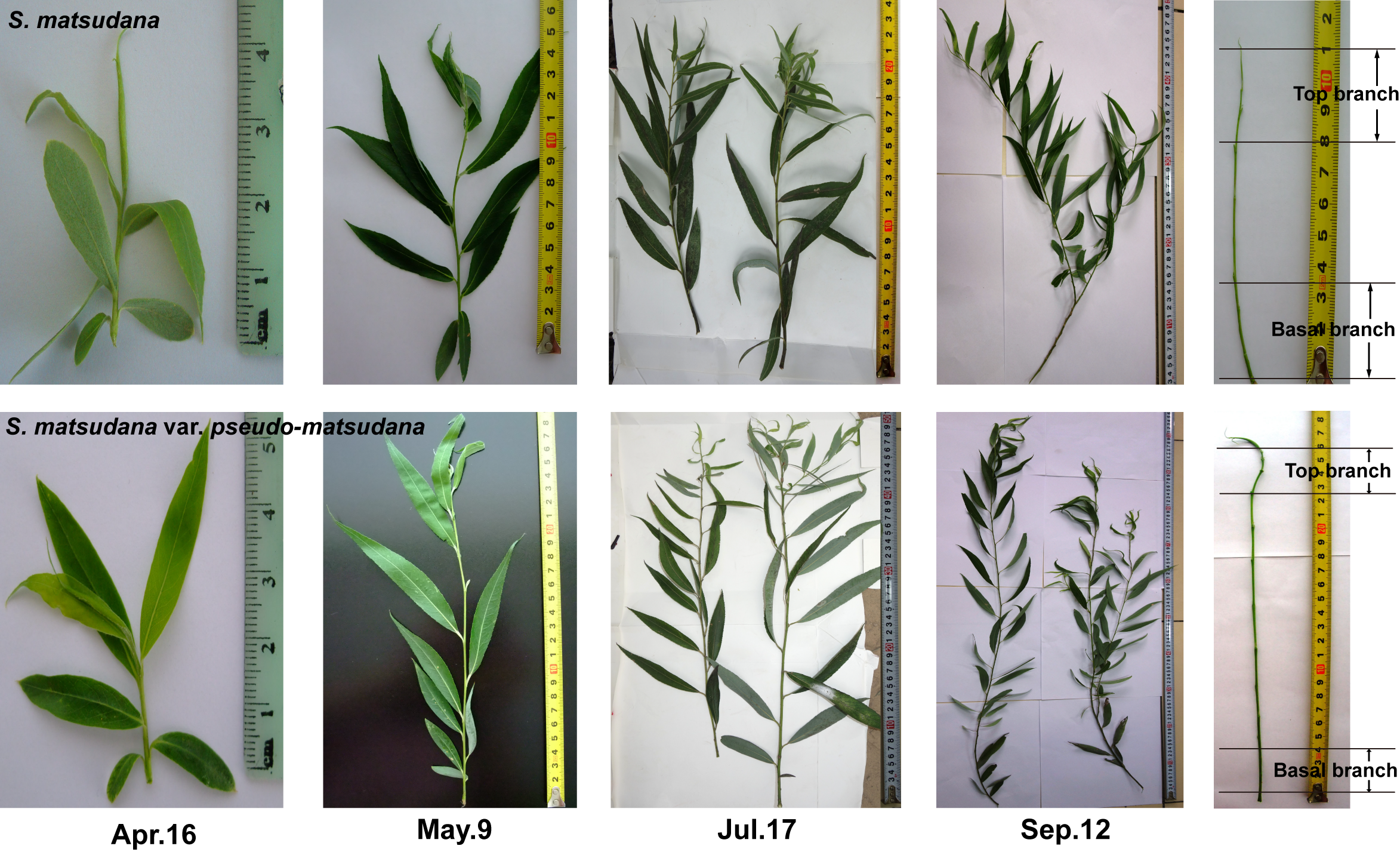

Supplement: Supplementary file 1 [file genes-08-00359-s001.zip › supplementary files/Figure S1.tif]

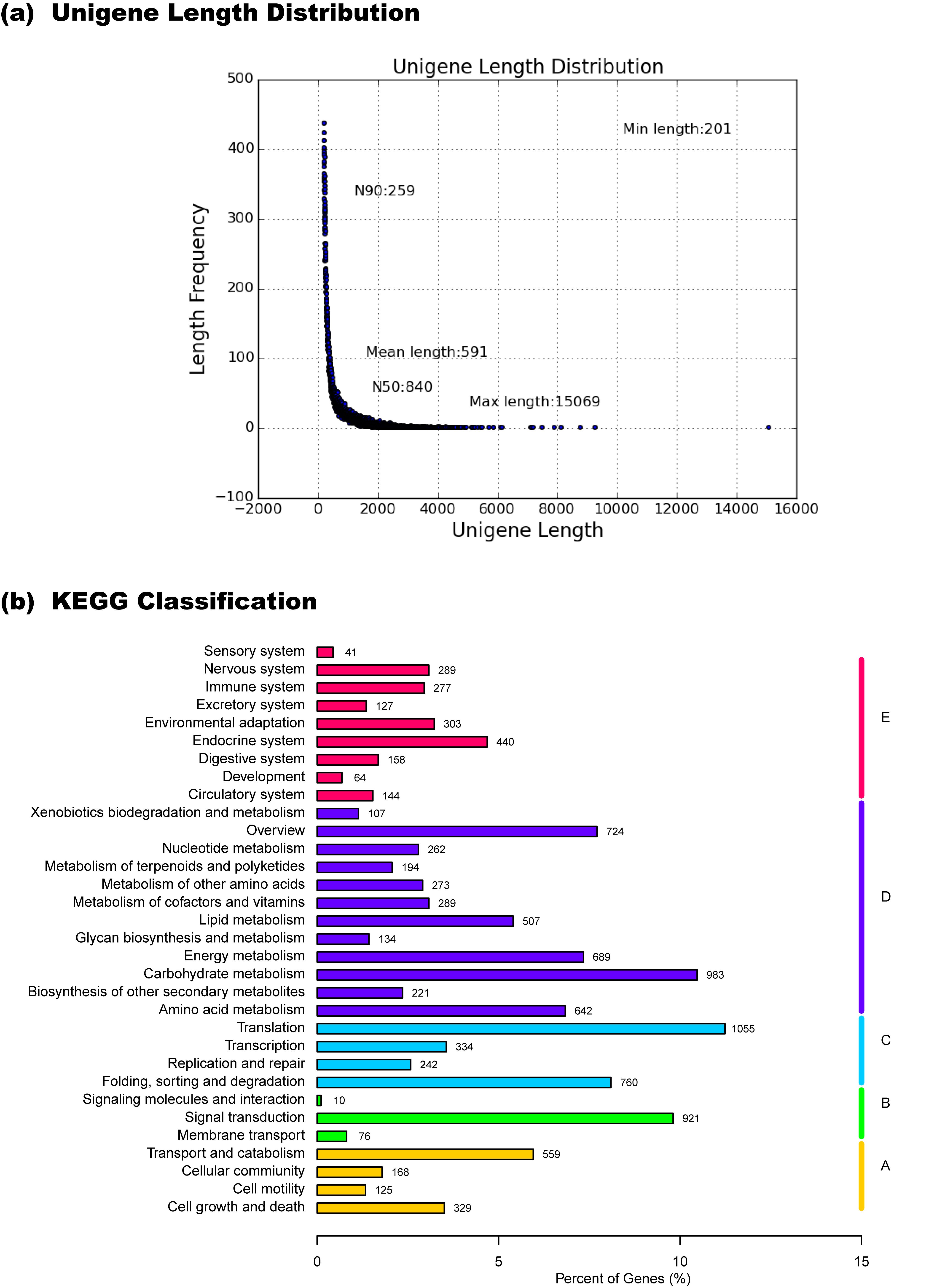

Supplement: Supplementary file 1 [file genes-08-00359-s001.zip › supplementary files/Figure S2.tif]

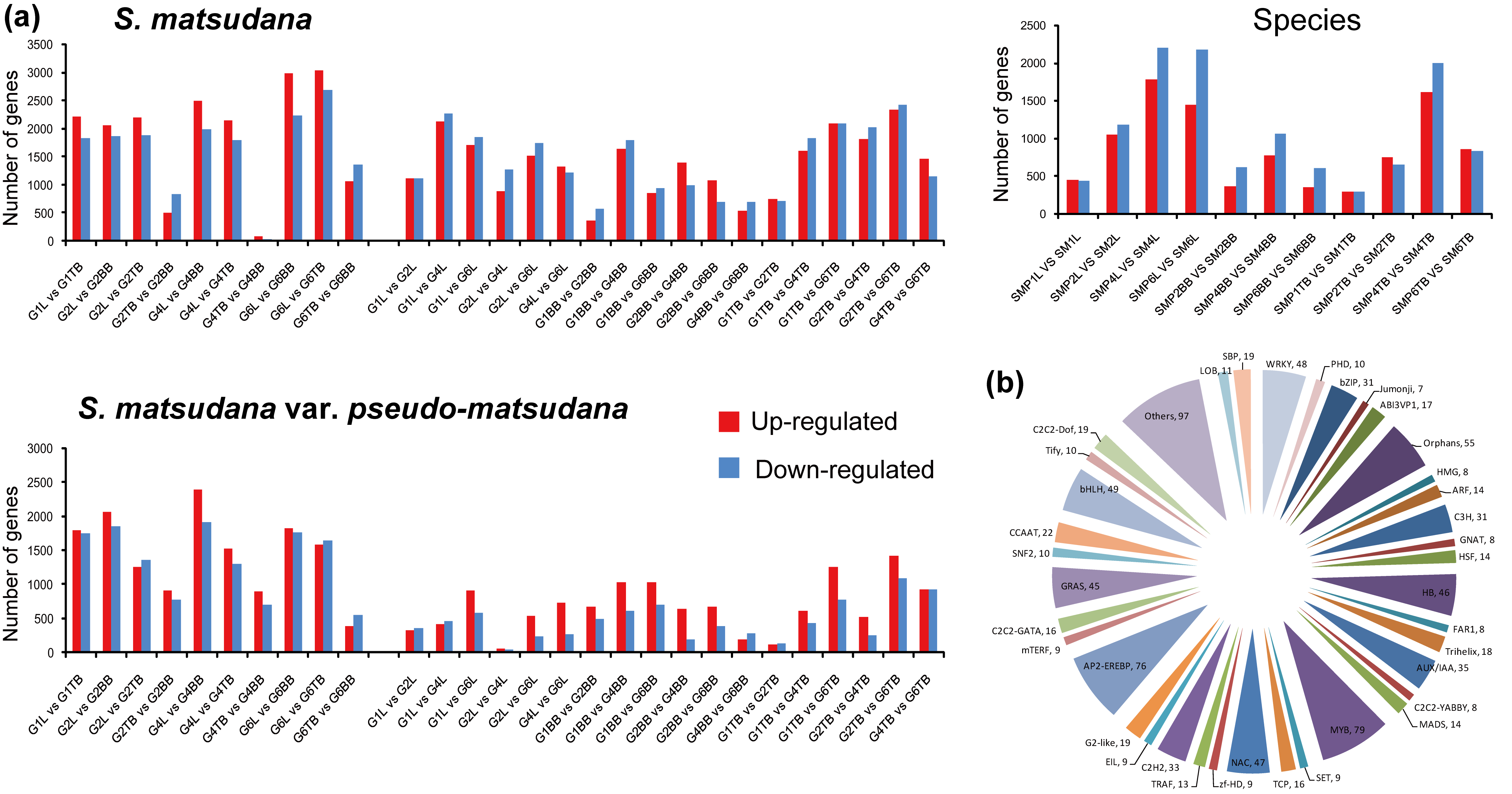

Supplement: Supplementary file 1 [file genes-08-00359-s001.zip › supplementary files/Figure S3.tif]

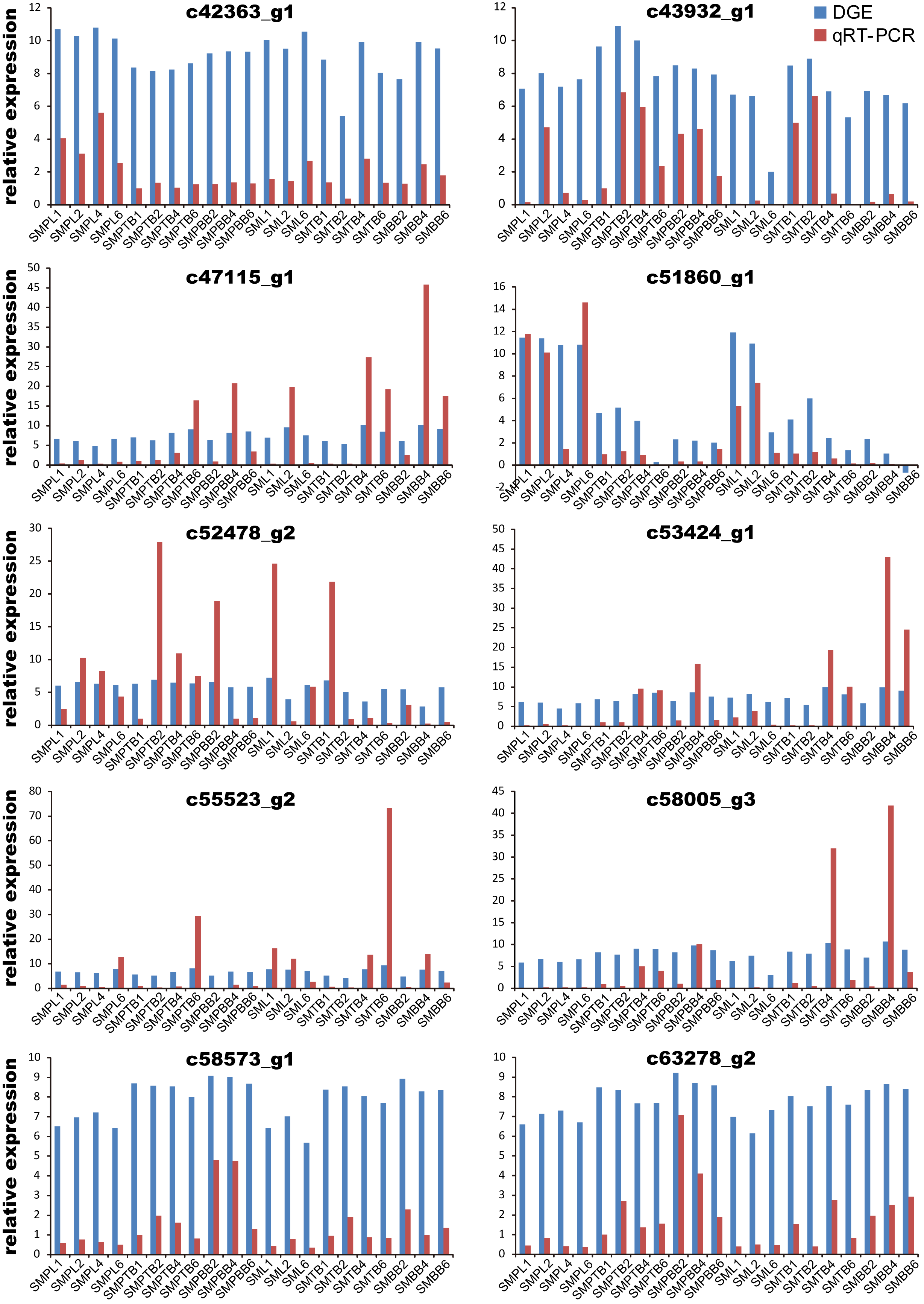

Supplement: Supplementary file 1 [file genes-08-00359-s001.zip › supplementary files/Figure S4.tif]
