# Supplementary material for: Transcriptional and Hormonal Regulation of Weeping Trait in Salix matsudana
Source: Genes (Basel). 2017 Nov 30;8(12):359. doi: 10.3390/genes8120359 (PMC5748677; doi:10.3390/genes8120359)
Supplement: Supplementary file 1 [file genes-08-00359-s001.zip › supplementary files/Table S1.doc]

**Table S1. Primers used for qRT-PCR.**

| Gene ID | Primer Name | Sequence 5’-3’ |
| --- | --- | --- |
| c42363_g1 | 96-1>c42363_g1-F | GTGTGGTGAAGAGGCACGAG |
|  | 96-1>c42363_g1-R | TGCTGAGGCCATTTTTTCTG |
| c43932_g1 | 96-2>c43932_g1-F | TCCTGGCTCTCATTGCCATT |
|  | 96-2>c43932_g1-R | AAGCTCTTGAGACTCCCCGG |
| c47115_g1 | 96-3>c47115_g1-F | TGGACTCGAAAATGGCCAAG |
|  | 96-3>c47115_g1-R | AAGGTGGTCACGTTGGTGGA |
| c51860_g1 | 96-4>c51860_g1-F | CATGGCTCGTTTGGACTTGG |
|  | 96-4>c51860_g1-R | CGGCACAGATTGACCCAGAG |
| c52478_g2 | 96-5>c52478_g2-F | TTCCATAGGAATCGACCCGC |
|  | 96-5>c52478_g2-R | CCTGAGATGCCTGCAGCAAG |
| c53424_g1 | 96-6>c53424_g1-F | AGGATGCAACTTTTTCCCGC |
|  | 96-6>c53424_g1-R | GGCCGTTGCCTTACACCTCT |
| c55523_g2 | 96-7>c55523_g2-F | GTTGTTTTGCTGCCACCACC |
|  | 96-7>c55523_g2-R | AATCTGTGTGCAGCTTCGGC |
| c58005_g3 | 96-8>c58005_g3-F | CCCATGAGTGCCGCATTTAT |
|  | 96-8>c58005_g3-R | TCGTAGAGCTGGCCTCGAAG |
| c58573_g1 | 96-9>c58573_g1-F | ACTGAGACCCAGGAAGCCCA |
|  | 96-9>c58573_g1-R | TTCCGTGGACAGCTCGACAG |
| c63278_g2 | 96-10>c63278_g2-F | AGAAGTGTTGGCGCGGTATG |
|  | 96-10>c63278_g2-R | GCGAGTATCCTCAATGCCCC |
| c57643_g1 | 96-11>c57643_g1-F | GTAACGGCAGCAGCAACTCG |
|  | 96-11>c57643_g1-R | GGCGGAGCTTGATCTGGAAC |
